# Evaluation of risk factors for obstruction of the intraventricular catheter after ventriculoperitoneal shunting in dogs with congenital internal hydrocephalus

**DOI:** 10.3389/fvets.2025.1688849

**Published:** 2026-01-08

**Authors:** Anna K. Siwicka, Daniela Farke, Kathrin Büttner, Andreas Moritz, Martin J. Schmidt

**Affiliations:** 1 Department of Veterinary Clinical Sciences, Small Animal Clinic, Justus Liebig University, Giessen, Germany; 2 Unit for Biomathematics and Data Processing, Faculty of Veterinary Medicine, Justus Liebig University Giessen, Germany

**Keywords:** hydrocephalus, obstruction, catheter, canine, magnetic resonance imaging, cerebrospinal fluid

## Abstract

**Background:**

Ventriculoperitoneal shunting (VPS) is the standard treatment for congenital internal hydrocephalus in dogs; however, obstruction of the ventricular catheter is a frequent and serious complication. In human medicine, several predictors of catheter obstruction have been identified, while corresponding risk factors in veterinary patients remain largely unknown.

**Objectives:**

This study aimed to identify potential risk factors for ventricular catheter obstruction after VPS implantation in dogs with congenital internal hydrocephalus.

**Methods:**

A retrospective cohort study was conducted involving 100 client-owned dogs diagnosed with congenital internal hydrocephalus and treated with VPS implantation between 2001 and 2022. The medical records and magnetic resonance imaging (MRI) data of these dogs were reviewed for age, sex, breed, body weight, ventricular size, ventricular catheter position, and preoperative medical treatments. Cerebrospinal fluid (CSF) samples were analyzed for cell count, red blood cell count, protein concentration, and cytology before and after surgery. Single logistic regression and chi-squared tests were performed to evaluate associations with ventricular catheter obstruction.

**Results:**

Obstruction occurred in 9 of the 100 dogs examined (9%; 95% confidence interval 3–15%), with an onset ranging from 8 to 210 days after VPS surgery (median 38 days). No significant associations were found between demographic factors, ventricle-to-brain ratio, ventricular catheter position, preoperative medical treatment, or CSF parameters and the occurrence of obstruction. Cerebrospinal fluid analysis revealed marked postoperative increases in cell counts, protein concentration, and red blood cell counts, peaking within the first week after surgery and gradually returning to reference ranges at the 3-month follow-up. Histological examination of obstructed catheters in three dogs demonstrated intraluminal blockage caused by inflammatory infiltrates, fibrin, and choroid plexus cells.

**Conclusion:**

Obstruction of the ventricular catheter remains a clinically important complication after VPS implantation in dogs with internal hydrocephalus. No definitive risk factors could be identified in this study. The observed postoperative CSF changes appear to reflect inflammatory and surgical responses rather than being predictors of obstruction. Further investigations with larger case numbers and detailed immunological analyses are required to clarify the underlying mechanisms and to improve prevention strategies.

## Introduction

Implantation of a ventriculoperitoneal shunt (VPS) system remains the treatment of choice in dogs with internal hydrocephalus, offering reconstitution of the brain parenchyma and resolution of clinical signs (1–3). However, complications such as obstruction of the ventricular catheter resulting in shunt failure within the first 3–6 months after VPS implantation can occur (1, 2). The etiologies of ventricular catheter obstruction are largely unknown in animals.

In children, shunt failure occurs in up to 30–50% of patients, with obstruction of the ventricular catheter being responsible for half of these failures (4–7). Different etiologies have been described in children with internal hydrocephalus, and obstruction rates vary between them (8). Ventricular catheter obstruction is most frequently observed in children with post-hemorrhagic internal hydrocephalus (8). After a VPS is implanted in the brain and its ventricular system, there is evidence that the brain shows an immunological response characterized by the recruitment of activated microglia, macrophages, neutrophils, and lymphocytes, which are involved in foreign body responses and inflammation around the catheter (9–13). This also includes vascular damage and astrogliosis, which changes the local environment surrounding the shunt (10–13). Other potential causes for the obstruction of a ventricular catheter in humans are the adhesion of choroid plexus cells along the catheter and malpositioning of the catheter within the brain parenchyma or ventricular wall (14, 15). Predictors of obstruction in human medicine include younger age at surgery, a long duration of the catheter lying *in situ* within the ventricle, and contact between the ventricular catheter and the ventricular wall. Furthermore, the number of holes at the tip of the ventricular catheter has been shown to be correlated with an increased need for revision surgeries in human medicine (15). However, risk factors for obstruction have not been described in animals. This study aimed to evaluate risk factors for ventricular catheter obstruction leading to subsequent malfunction of the shunt system.

## Materials and methods

In this retrospective cohort study, the databases of the Department of Veterinary Clinical Science, Small Animal Clinic, Justus Liebig University Giessen, Germany, were searched for records of animals diagnosed with internal hydrocephalus and treated with the implantation of a VPS between January 2001 and June 2022. The diagnosis of congenital internal hydrocephalus was based on magnetic resonance imaging (MRI) findings consistent with ventriculomegaly and parenchymal thinning, along with clinical indications for VPS surgery due to neurological symptoms consistent with forebrain or brainstem localization (16, 17). Data collected from the records included the animals’ age in months at the time of diagnosis, breed, sex, body weight, neutering status, and MRI findings before and after VPS implantation. Cerebrospinal fluid was collected before surgery or during surgery and every 2 days after surgery for a period of approximately 1 week during hospitalization. The cerebrospinal fluid was analyzed for cell counts (cells/μL), red blood cell counts (RBCs) (cells/μL), and protein concentration (mg/L), and a qualitative cytological assessment was performed. Obstruction of the ventricular catheter was diagnosed if the CSF reservoir did not refill after manual compression. A follow-up MRI was performed after 3 months if any deterioration in the clinical signs was noted. All dogs with a diagnosis of congenital internal hydrocephalus confirmed by MRI and treated with VPS implantation during the study period were eligible.

### Magnetic resonance imaging

Imaging was performed using a 1.5 Tesla high-field MRI scanner (Siemens Verio, Siemens Healthcare, Erlangen, Germany) before 2017, and a 3.0 Tesla high-field MRI scanner (Philips Intera Gyroscan, Philips Healthcare, Hamburg, Germany) between 2017 and 2022. The images included at least sagittal, transverse, and dorsal T2-weighted images (Turbo Spin Echo, TR 2900 ms, TE 120 ms, slice thickness of 3 mm) and T1-weighted pre- and post-contrast medium administered images (TR 588, TE15, slice thickness of 1 mm).

### Image analysis

All MRI datasets were retrieved from the relevant picture archiving and communication system and evaluated retrospectively by a board-certified neurologist (DF). The images were obtained before VPS surgery, at a 3-month follow-up for routine postoperative evaluation, or after deterioration of clinical signs. They were evaluated for the ventricle-to-brain ratio (VBR) and the position of the tip of the ventricular catheter. The ventricle-to-brain ratio was measured on dorsal T2-weighted MR images at the widest point of the lateral ventricles. The maximal width between the lateral borders of both ventricles was divided by the maximal internal width of the brain at the same level (16, 17).

The tip of the ventricular catheter contained multiple holes over a length of 1.5 cm. The position of the ventricular catheter was defined as follows: (1) inside the ventricle, if at least 1.5 cm of the catheter was within the ventricular lumen; (2) partially within the brain parenchyma, if less than 1.5 cm of the catheter was inside the ventricular lumen; and (3) completely inside the parenchyma, if the catheter did not penetrate the ventricular lumen at all.

### Shunting procedure and cerebrospinal fluid sampling

**Figure 1:**
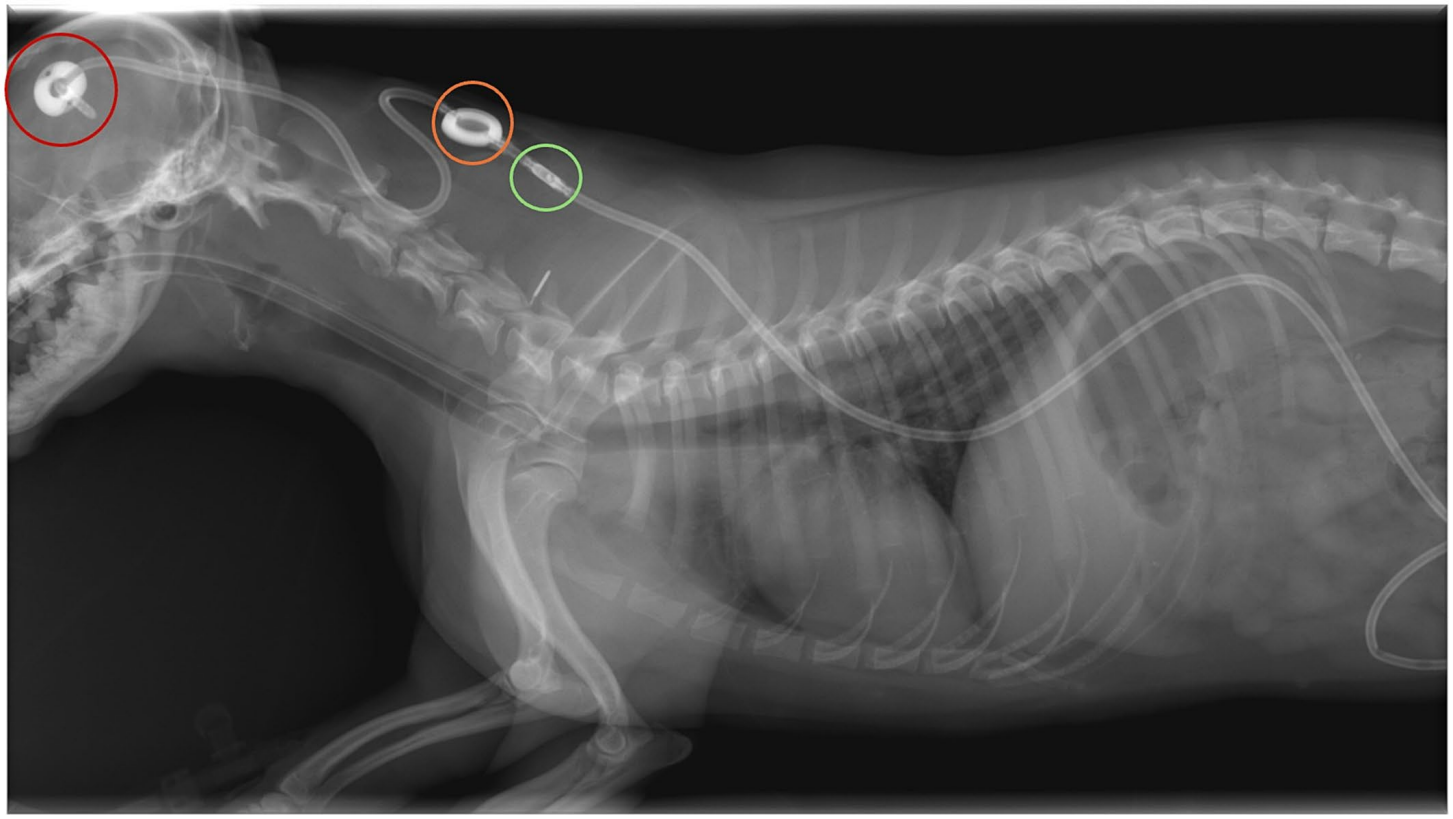
Lateral radiograph of a dog following ventriculoperitoneal shunt placement. The integrated reservoir (orange circle) and differential pressure valve (green circle) are visible as a radiopaques structures over the dorsal cervical soft tissues. The deflector is indicated by the red circle.

All dogs were premedicated with a benzodiazepine and an opioid (most commonly fentanyl, administered as a bolus followed by a continuous rate infusion during surgery). Anesthesia was induced with alfaxalone in the majority of cases and maintained with inhalational isoflurane in oxygen and air. Animals were placed in right lateral recumbency without elevation of the head. A 0.7-cm diameter craniotomy was drilled into the left parietal bone of each animal. The appropriate insertion depth of the ventricular catheter was determined preoperatively by measuring the distance from the skull surface to the midline of the ventricle on MRI images. The vessels of the dura mater were cauterized, and a blade incision was performed to open the dura mater. Ventriculoperitoneal shunt systems with an integrated CSF reservoir and pressure valves were used for surgery in all dogs (MiniNav, MIETHKE^®^ Potsdam, Germany). The reservoir was placed subcutaneously in the cervical region to allow repeated percutaneous CSF access for monitoring (Figure 1). The type of pressure valve (5, 10, or 15 mmHg) was selected based on an intraoperative measurement of intracranial pressure. The ventricular catheter was inserted into the lateral ventricle through the drill hole, and CSF flow was visible through the catheter. The system is equipped with a deflector that allows the catheter to exit the drill hole at a right angle without kinking. This component is positioned on the outer surface of the skull. Long-term stabilization of the proximal catheter segment is achieved through the surrounding soft tissues, particularly the temporal musculature, and subsequent fibrous tissue formation around the implant. The remainder of the catheter was tunneled subcutaneously using a needle trocar and placed in several loops to allow for movement and growth. The abdominal catheter was placed into the abdominal cavity via an approach through the abdominal wall, two fingers behind the last ribs, and fixed using a Chinese finger trap suture with Ethilon 3.0. Postoperative care included analgesia with oral metamizole (50 mg/kg every 8h) and intravenous methadone (0.1 mg/kg every 4 h), along with antibiotic treatment with intravenous cefotaxime (20 mg/kg every 8 h). The first CSF sample was taken during surgery. Further samples were taken subcutaneously after surgery during hospitalization and after 3 months, using the CSF reservoir that was integrated into every VPS system. In total, revision surgery was performed on all nine dogs diagnosed with ventricular catheter obstruction. One 3.3-month-old dog showed obstruction of the ventricular catheter 17 days after VPS surgery and 10 days after revision surgery. Revision surgery was performed twice, and histopathological examination was performed on each ventricular catheter. In total, histopathological examination of obstructed ventricular catheters was performed in three dogs.

#### Cerebrospinal fluid analysis

Cerebrospinal fluid was sampled in plain tubes and processed within 30 min after collection. Cerebrospinal fluid cell count (cells/μL), RBC count (cells/μL), protein concentration (mg/L), and CSF cytology of each CSF sample were evaluated. Cell counts were measured using a flow cytometry-based analysis using an Advia 2120i (Siemens Healthineers AG, Forchheim, Germany), and protein concentration was measured using an ABX Pentra 400 (Horiba ABX SAS Europe Montpellier, France). Cytological evaluation was performed manually by a board-certified veterinary clinical pathologist using a microscope (Olympus CX31, EVIDENT Europe GmbH, Hamburg, Germany).

Approval from the ethics committee of the Justus Liebig University was not sought, as retrospective studies of images and records stored in the archive are not subject to ethical review.

### Statistical analysis

Statistical analysis was performed using a commercial statistical software package (Base SAS^®^ 9.4 Procedures Guide: Statistical Procedures, Second Edition, Statistical Analysis System Institute Inc., Cary, NC, USA). The prevalence of obstruction was assessed, and a Wald confidence interval (CI) of 95% was determined. The complications of obstruction were evaluated as a dependent, dichotomous variable. VBR before surgery, and maximal values of CSF cell count (cells/μL), RBC count (cells/μL), and CSF protein concentration (mg/L) over time were evaluated as independent variables to assess their influence on obstruction of the ventricular catheter using a single logistic regression analysis. An exact Pearson’s chi-squared test was performed to assess the influence of ventricular catheter position and medical treatment before surgery as possible risk factors for ventricular catheter obstruction. For all statistical tests, a significance level of 0.05 was applied. Because these variables were selected *a priori* based on biological relevance, the *p*-values were not adjusted for multiple comparisons.

The course of CSF cell count, CSF protein concentration, and RBC count, in addition to cytological findings over time, was analyzed descriptively. Data were presented with a median and range.

## Results

### Animals

A total of 100 dogs were included in the study. The most common breeds were Chihuahua (*n* = 25), mixed-breed dogs (*n* = 10), Labrador Retriever (*n* = 6), French Bulldog (*n* = 5), Maltese (*n* = 5), and Yorkshire Terrier (*n* = 3). Other breeds were represented by one or two dogs each. The median age was 0.9 months (0.06–65.3 months), and the median body weight was 4.5 kg (1–58 kg). Of the dogs, 37 were intact females, 6 were neutered females, 47 were intact males, and 10 were neutered males. All dogs were evaluated for body weight, age, and sex. No apparent associations between body weight, age, or sex and the obstruction of the ventricular catheter were found.

Nine animals presented with obstruction of the ventricular catheter, occurring across several different breeds (Bull Terrier, Labrador Retriever, Chihuahua, French Bulldog, and mixed-breed dogs). The time taken for neurological status to deteriorate due to obstruction of the ventricular catheter ranged from 8 to 210 days (median 38 days) after VPS surgery. The prevalence of obstruction of the ventricular catheter and associated malfunction of the VPS system was 9% (CI 95% 3–15%). In dogs with an obstruction of a ventricular catheter, owners requested euthanasia in two dogs shortly after revision surgery due to progressive deterioration.

In total, postoperative complications were observed in 30% (30/100) of dogs. Ventricular catheter obstruction accounted for 30% (9/30) of all complications. The remaining 70% (21 dogs) experienced other postoperative complications ranging from minor events such as catheter kinking or disconnection to major complications, including ventricular collapse or hemorrhage.

### Risk factor evaluation

#### Risk factor evaluation—pretreatment

A total of 42 dogs were pretreated with various medications, including prednisolone at dosages ranging from 0.5 to 2 mg/kg once daily in 34 dogs, omeprazole (1mg/kg once daily) in 23 dogs, furosemide (2 mg/kg once daily in 9 dogs), and acetazolamide (10 mg/kg 3 times a day) in 4 dogs. Treatment combinations included prednisolone and omeprazole in 17 dogs, prednisolone and furosemide in 4 dogs, and prednisolone and acetazolamide in 2 dogs. Prednisolone, omeprazole, and furosemide were given together to 3 dogs. None of these treatments was associated with ventricular catheter obstruction (*p* = 0.2233).

#### Risk factor evaluation—MRI findings

The median VBR before surgery was 0.79 (0.6–0.95) in dogs without ventricular catheter obstruction and 0.70 (range 0.63–0.95) in dogs with obstruction. No associations were found between the VBR and the obstruction of the ventricular catheter (*p* = 0.5741).

An MRI examination after 3 months was performed in 78 dogs. The ventricular catheter was completely placed within the ventricle (1) in 22 dogs, partially within the parenchyma (2) in 42 dogs, and completely within the parenchyma (3) in 14 dogs. These positions were evaluated independently, and another evaluation was performed summarizing the partial and complete positioning of the ventricular catheter inside the brain parenchyma. No statistically significant association of ventricular catheter positioning and its obstruction could be identified (*p* = 0.3182).

#### Risk factor evaluation—CSF

**Figure 2:**
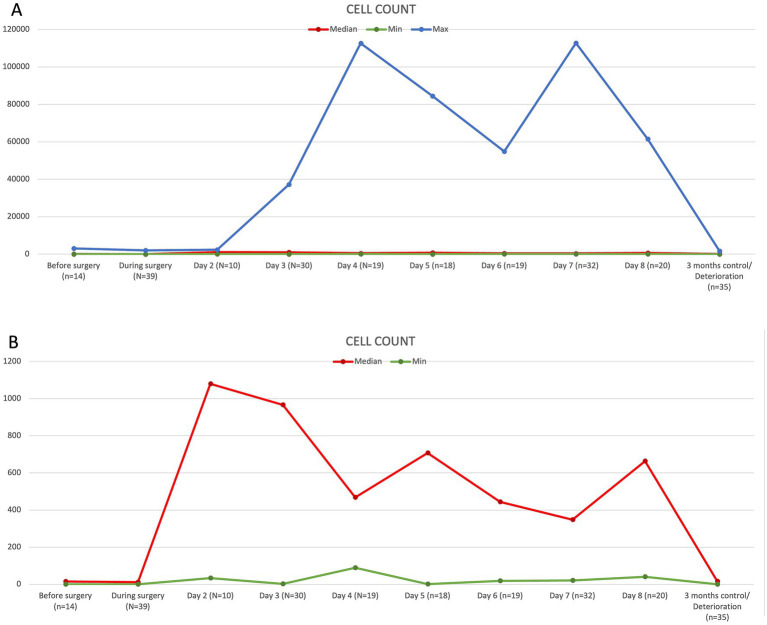
Graphic demonstration of the development of the CSF cell count in cells/μL over time. The upper graph **(A)** shows minimal (green), median (red), and maximal (blue) values. The lower graph **(B)** compares minimal (green) and median (red) values only.

Cerebrospinal fluid measurements were collected at various time points during a hospitalization of 1–8 days and at a 3-month follow-up. CSF samples were not available at each time point for every animal. Cerebrospinal fluid cell counts before surgery and intraoperatively were low in all measured animals, with a median of 15 cells/μL (1–2,073 cells/μL) and 12 cells/μL (1–3,072 cells/μL). On the second day after surgery, the median cell count was 1,079 cells/μL (34–2,356 cells/μL). There was a gradual decline in CSF cell count, with small peak cell concentrations at days 5 and 8 after surgery and the lowest CSF cell counts at 3 months after surgery, with a median of 17 cells/μL (1–1,647 cells/μL) (Figure 2).

**Figure 3:**
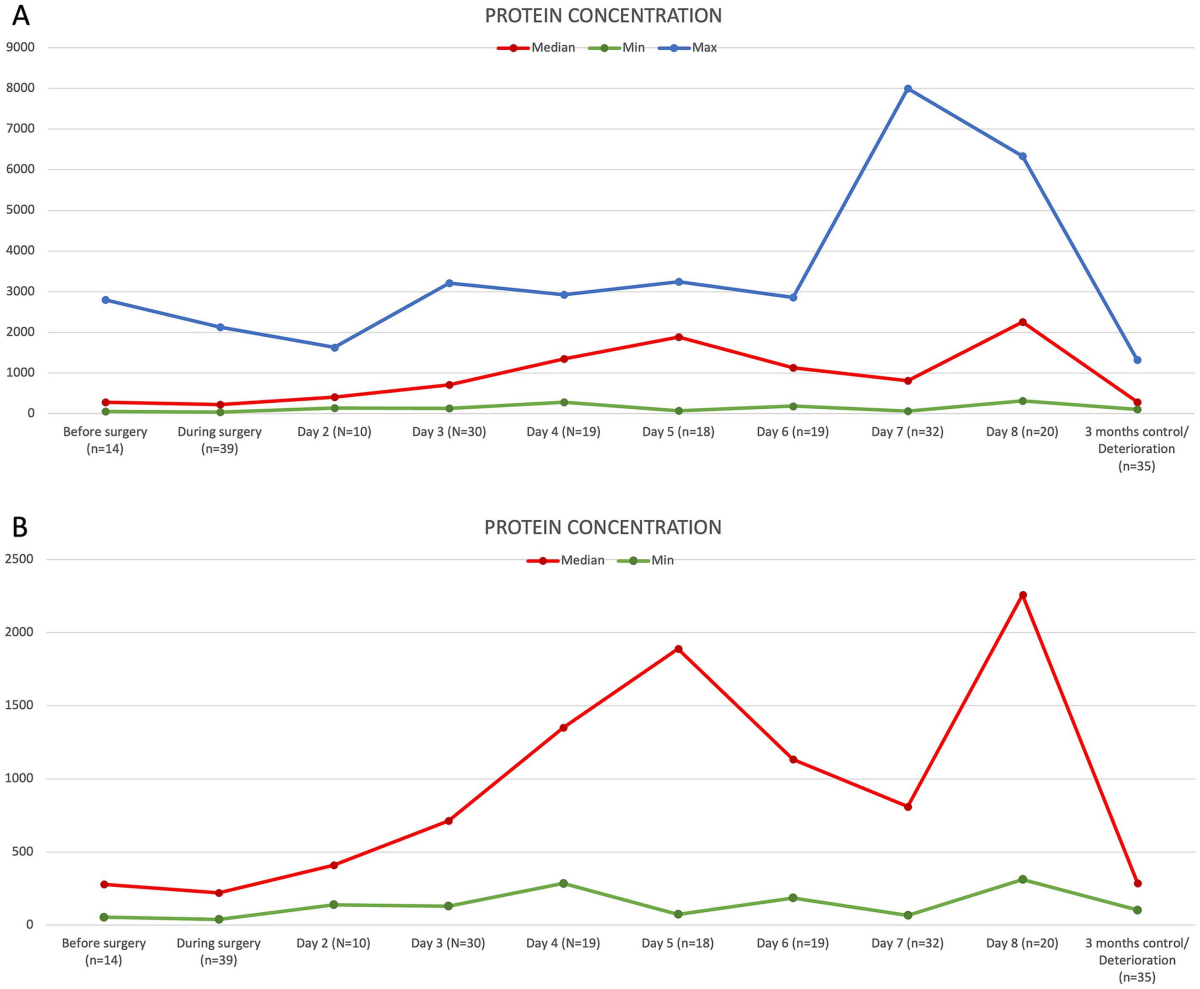
Graphic demonstration of the development of the protein concentration in mg/L over time. The upper graph **(A)** shows minimal (green), median (red), and maximal (blue) values. The lower graph **(B)** compares minimal (green) and median (red) values only.

CSF protein levels before and during surgery were measured in the majority of dogs, with medians of 279.8 mg/L and 220.8 mg/L, respectively. Two peak protein concentrations were observed at day 5 after surgery, with a median of 1,889.3 mg/L (73.6–3,248.3 mg/L), and at day 8, with a median of 2,259.9 mg/L (range 312.6–6,335.9 mg/L). The lowest CSF protein level was observed at 3 months post-surgery, with a median of 286.9 mg/L (103.4–1,328.3 mg/L) (Figure 3).

**Figure 4:**
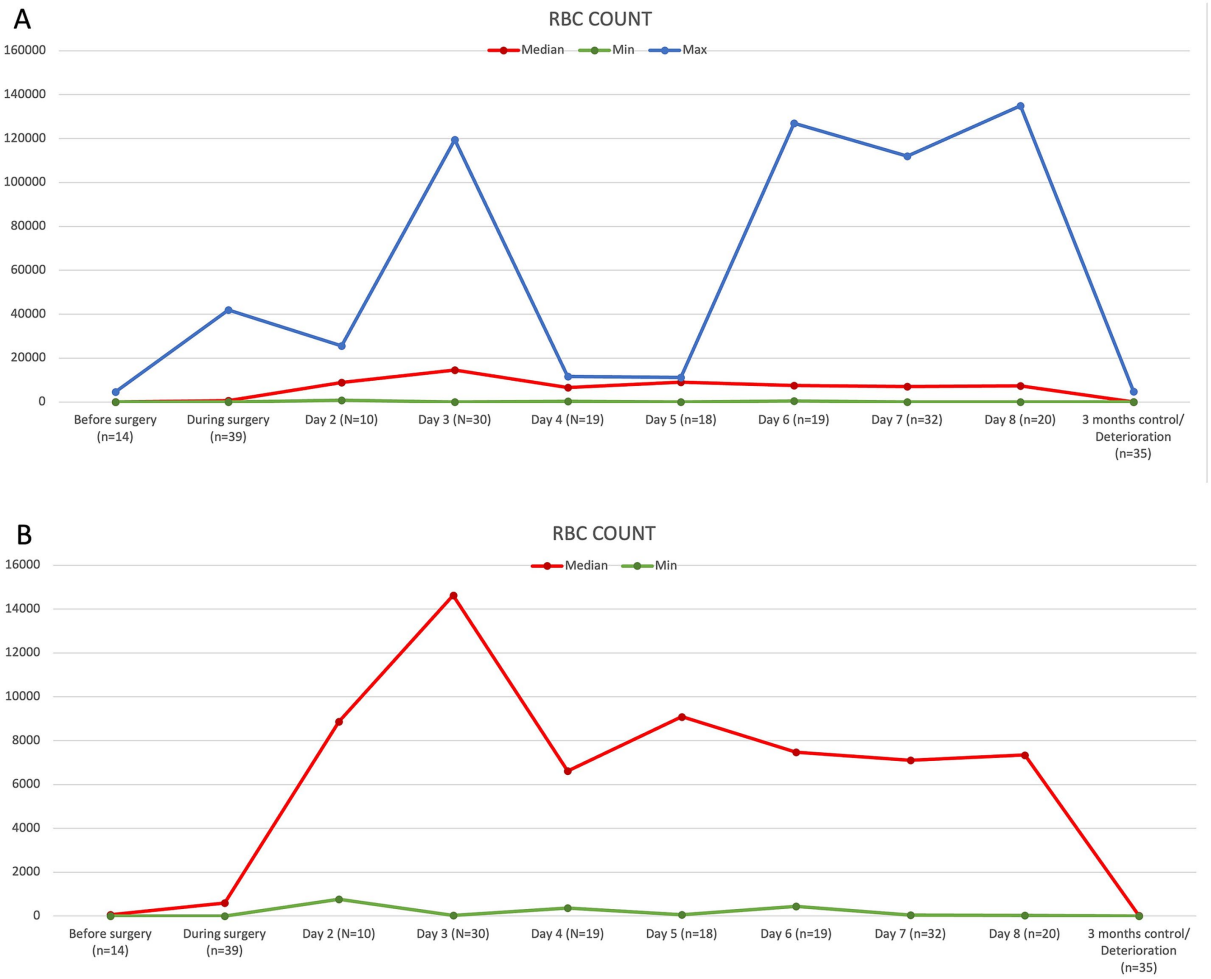
Graphic demonstration of the development of the RBC count in cells/μL over time. The upper graph **(A)** shows minimal (green), median (red), and maximal (blue) values. The lower graph **(B)** compares minimal (green) and median (red) values only.

RBC contamination and xanthochromia were routinely observed after VPS placement. The median RBC count reached its highest level on day 3 after surgery, with a median of 14,623 cells/μL (23–119,582 cells/μL), and then declined over time, with the lowest median value of 8 cells/μL (0–4,709 cells/μL) observed at the 3-month follow-up (Figure 4). A summary of all CSF cell count, RBC count, and protein concentration values is provided in Table 1.

**Table 1:** Summary of median, minimal, and maximal values of protein concentrations in mg/L, RBC counts in cells/μL and CSF cell counts in cells/μL over time.

CSF	Before surgery	*n* = 14		During surgery	*n* = 39		Day 2	*n* = 10		Day 3	*n* = 30		Day 4	*n* = 19		Day 5	*n* = 18		Day 6	*n* = 19		Day 7	*n* = 32		Day 8	*n* = 20		3 months	*n* = 35	
	Protein	RBC	Cells	Protein	RBC	Cells	Protein	RBC	Cells	Protein	RBC	Cells	Protein	RBC	Cells	Protein	RBC	Cells	Protein	RBC	Cells	Protein	RBC	Cells	Protein	RBC	Cells	Protein	RBC	Cells
Median	2,798	585	15	2,208	599	12	409	8,872	1,079	7,139	14,623	966	13,502	66,115	468	18,893	9,088	707	11,343	74,785	444	8,099	71,035	348	22,599	7,341	664	2,869	8	17
Min	54	0	1	391	3	1	1,391	765	34	1,298	23	3	2,857	367	89	736	54	2	1,858	444	19	668	48	21	312,6	33	41	1,034	0	1
Max	28,004	4,637	3,072	2,131,1	41,969	2,063	16,302	25,621	2,356	32,139	119,528	37,238	29,266	11,692	112,645	32,483	11,227	84,399	28,601	126,984	54,804	8,000	112,037	112,759	63,359	134,897	61,531	13,283	4,709	1,647

The number of observations evaluated is given for each time point (*n*).

The median maximal postoperative CSF cell count was 4,761.5 cells/μL (range 131–61,531) in dogs with obstruction and 752.5 cells/μL (range 2–112,759) in dogs without obstruction. The median maximal protein concentration was 2,017.8 mg/L (range 119.7–8,000) versus 1,134.3 mg/L (range 46–6,335.9), and the median maximal RBC count was 28,069 cells/μL (range 7,453–134,897) versus 9,702 cells/μL (range 3–119,528). Maximal CSF cell counts (*p* = 0.6761), RBC counts (*p* = 0.0878), and protein concentrations (*p* = 0.0718) were not significantly associated with the occurrence of ventricular catheter obstruction.

**Figure 5:**
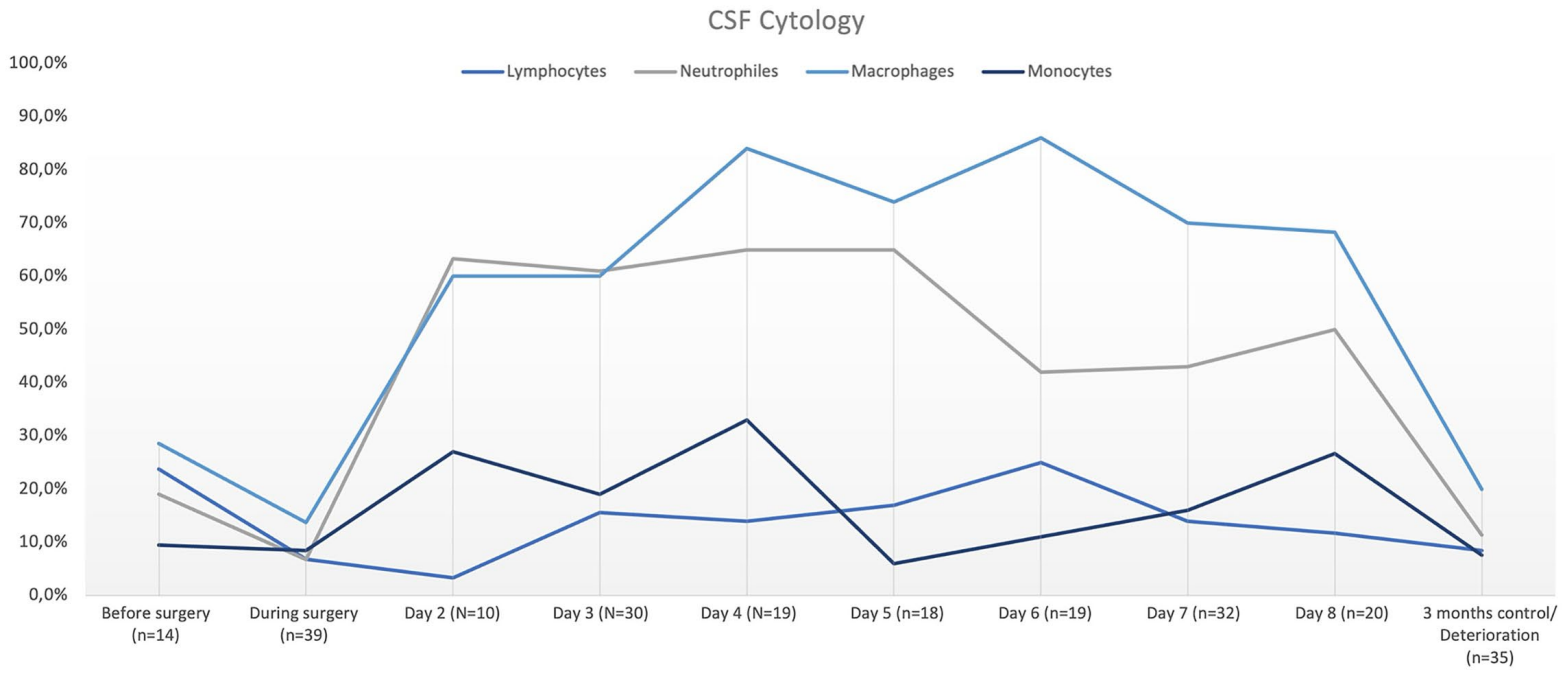
Graphic demonstration of CSF cytological findings in mean percentage over time after VPS surgery.

Qualitative cytological evaluation revealed overall mixed cell pleocytosis containing predominantly macrophages and neutrophils. Monocytes and lymphocytes were observed in smaller proportions (Figure 5).

**Figure 6:**
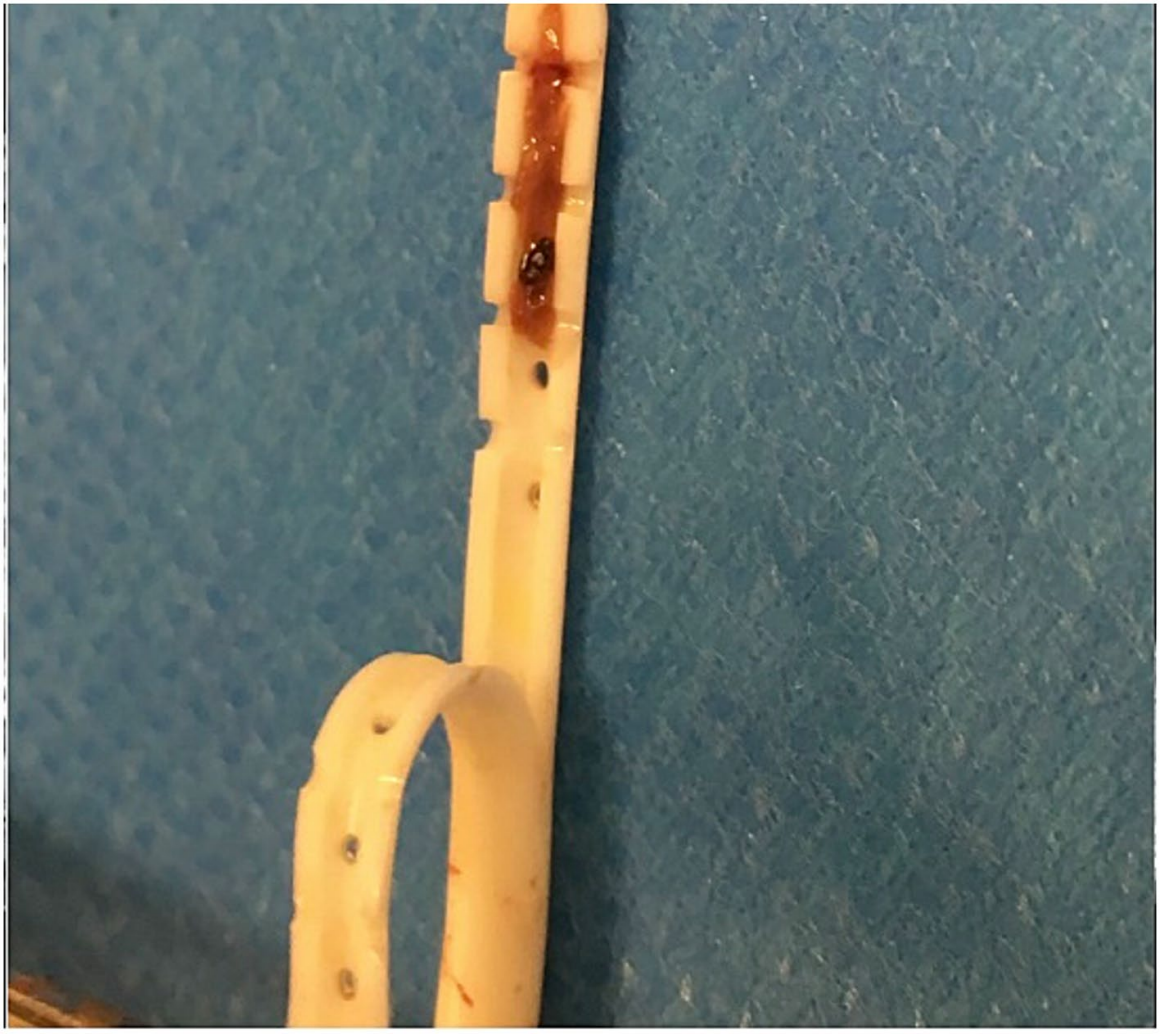
The ventricular catheter of a 3.3 months old dog that was removed during a revision surgery 17 days after initial implantation of the VPS. The catheter is cut in half at the length axis showing red to brownish material occluding the lumen at the tip and protruding into the perforations of the catheter.

Pathological examination of both ventricular catheters removed during revision surgery showed complete blockage of both catheters by brownish intraluminal material that protruded through the perforations of the catheters (Figure 6). Histopathological examination of the first catheter removed 17 days after the initial VPS implantation revealed highly vascularized granulation tissue, composed of degenerated neutrophils, a few macrophages, and fibrin components. Additionally, a few siderophages, dystrophic mineralization, and choroid plexus cells (stained positive for Goldner’s and Periodic acid–Schiff) were found. The histopathological findings of the second ventricular catheter that was removed 10 days following revision surgery showed partial astroglia and gitter cell invasion along with fibrin material, red blood cells, and a few neutrophils.

Not all catheters from the remaining eight dogs with obstruction were submitted for pathological examination. In those cases where histology was available, chronic inflammatory cell infiltration and partial astroglial proliferation were observed. In one dog, obstruction was associated with an intraluminal blood clot.

## Discussion

Despite good long-term outcomes in the majority of dogs, VPS continues to be associated with well-known complications (1, 2). The prevalence of obstruction was 9% (95% CI 3–15%) among the animals treated with VPS in this study. This rate is much lower than the complication rates reported in human medicine (4–7). This is most likely since etiologies differ among the species. Children are more often affected by posthemorrhagic and postinfectious internal hydrocephalus, which is more susceptible to ventricular catheter obstructions (8, 10). For dogs, these etiologies remain rare, and idiopathic causes without evidence of inflammation or hemorrhage are more common (1, 2). The systematic use of susceptibility-weighted studies aiming to identify periventricular hemorrhage in hydrocephalic animals would be valuable to evaluate this finding as a risk factor in the future. However, in this study, the RBC count concentration was low when measured before or during surgery, suggesting that a hemorrhagic etiology is not likely in dogs. RBC count was not associated with obstruction of the ventricular catheter in this study.

Concentrations of RBC, cells, and proteins were highest directly after surgery and then gradually declined. This is most likely explained by the disruption of small blood vessels during ventricular catheter insertion. Disruption of the ventricular lining may have allowed additional cells to enter the CSF, which likely explains the mixed pleocytosis found in the cytological assessment directly after surgery. The recruitment of inflammatory cells over time is also likely, as cell counts and protein concentrations showed different peaks on days 5 and 8 during hospitalization, whereas RBC counts showed a further decline with time. This would explain the large amounts of macrophages and neutrophils found in the CSF. The reason for the peaks in protein concentrations might also be related to inflammatory responses, such as elevations in cytokines. An increase in CSF cell counts concurrent with acute-phase proteins, such as C-reactive protein, or other inflammatory mediators, such as cytokines or immunoglobulins, that occur during inflammatory responses might also explain the simultaneous peaks in cell count and protein concentration observed in this study (18, 19). Time-dependent changes in the composition of the cell profile can be observed in humans after VPS implantation (7, 20). The differentiation is based on histopathological findings of intraluminal ventricular catheter tips, with samples divided into inflammatory-based reactions, based on the presence of lymphocytes, macrophages, and microglial cells, or reactive responses, based on the presence of fibroconnective tissue, reactive astrocytes, and Rosenthal fibers. The histopathological findings of the catheter tips examined in this study also revealed the involvement of the surrounding tissue and reactions, such as astroglial invasion and activation, or invasion of choroid plexus cells. Inflammatory reactions, including the involvement of neutrophils and macrophages, were also observed. This supports the theory that obstruction is caused by a combination of local tissue reactions, invasions, and inflammatory responses. However, this study primarily focused on CSF cell counts, so reactive responses of astrocytes and connective tissue as potential causes of obstruction were likely missed. Inflammatory responses, however, were observed in the CSF of all animals but could not be related to catheter obstruction. Further studies should therefore not only concentrate on histopathological evaluation of obstructed ventricular catheters, but also include a more detailed CSF examination of cell types, cytokines, and possible immunological profiles.

Deterioration of clinical signs occurred at a median of 38 days following surgery. This aligns with the reported timeframe for overall complications in dogs, occurring up to 6 months after surgery (1, 2). In humans, obstructions may occur early within the first month or at a later stage within 3 to 6 months (21). The duration of ventricular catheter placement was associated with the likelihood of obstruction in one study (15). This is believed to be a result of the reduction in ventricular size and subsequent contact of the ventricular catheter and the ependymal lining, or again due to an early inflammatory response, or because of a reactive tissue response at different time stages (15, 20, 21). In this study, the timeframe in which obstruction occurred ranged from 8 to 210 days. This relatively long time might be explained by different pathological mechanisms, which could also apply to animals.

The position of the ventricular catheter was completely within the lateral ventricle (22/78 dogs), partially within the parenchyma (42/78 dogs), or completely within the parenchyma (14/78 dogs). As already mentioned, contact between the perforated ventricular catheter tip and the ependymal lining of the ventricular wall is a known risk factor in humans (15). Invasion of local tissue astrocytes and parts of the choroid plexus was also evident in the histological examination of the catheter tips in this study; however, the position of the catheter could not be identified as a predisposing factor for obstructions in this study. Malfunction of the VPS in case of overshunting, whereby the brain parenchyma wraps around the tip of the ventricular catheter secondary to hemispheric collapse, has already been reported in dogs as a mechanical cause of obstruction (22). Special coatings and materials are designed for human medicine to reduce the adherence of local tissue, such as the choroid plexus, to the shunt material (23).

Given the retrospective character of our study and the single logistic regression analysis of each variable, we cannot exclude bias. Influences from one variable on another cannot be excluded completely for risk factor evaluation, such as CSF parameters. The main limitation of this study is the low number of obstructions observed. Although we had a large sample size, the prevalence of obstruction was very low, which most likely explains the low significance of the risk factor analysis. A larger sample size could have been achieved by including more than one facility to conduct a multicenter study. Another limitation of the study is that not all CSF parameters could be analyzed for all patients and time points after surgery. Data were missing due to several circumstances, such as the animal’s temperament, owner compliance, limited laboratory assessment during weekends, and a limited amount of CSF that could be evacuated from the reservoir. Due to the heterogeneous distribution and small case numbers within individual breeds, no statistical evaluation could be conducted, and no apparent breed predisposition was observed. Further studies should include more cases of obstructions with consequent follow-up of CSF taps. Although ventricular catheter position could not be identified as a cause of obstruction, histopathological analysis of the ventricular catheter tip in these cases is warranted to determine the cause of obstruction and the underlying pathomechanisms.

## Conclusion

Obstruction of the ventricular catheter remains a severe complication after VPS placement in dogs with internal hydrocephalus. No risk factors could be identified in this study. Cerebrospinal fluid cell counts and protein concentrations are severely elevated after VPS implantation and gradually decrease to normal values over time.

## Data Availability

The raw data supporting the conclusions of this article will be made available by the authors, without undue reservation.
