# Supplementary figures and images for: Evaluation of risk factors for obstruction of the intraventricular catheter after ventriculoperitoneal shunting in dogs with congenital internal hydrocephalus
Source: Front Vet Sci. 2026 Jan 8;12:1688849. doi: 10.3389/fvets.2025.1688849 (PMC12824531; doi:10.3389/fvets.2025.1688849)

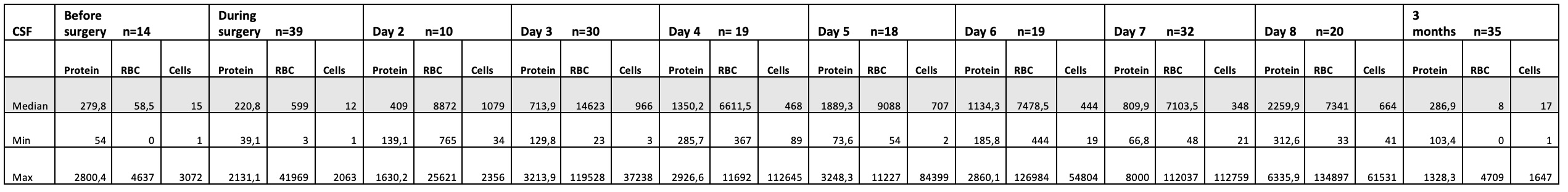

Supplement: Supplementary file 1 [file Image_1.jpeg]
